# Reliability of Transcriptional Cycles and the Yeast Cell-Cycle Oscillator

**DOI:** 10.1371/journal.pcbi.1000842

**Published:** 2010-07-08

**Authors:** Volkan Sevim, Xinwei Gong, Joshua E. S. Socolar

**Affiliations:** 1 Physics Department and Center for Nonlinear and Complex Systems, Duke University, Durham, North Carolina, United States of America; 2 Center for Systems Biology, Institute for Genome Science and Policy, Duke University, Durham, North Carolina, United States of America; Pennsylvania State University, United States of America

## Abstract

A recently published transcriptional oscillator associated with the yeast cell cycle provides clues and raises questions about the mechanisms underlying autonomous cyclic processes in cells. Unlike other biological and synthetic oscillatory networks in the literature, this one does not seem to rely on a constitutive signal or positive auto-regulation, but rather to operate through stable transmission of a pulse on a slow positive feedback loop that determines its period. We construct a continuous-time Boolean model of this network, which permits the modeling of noise through small fluctuations in the timing of events, and show that it can sustain stable oscillations. Analysis of simpler network models shows how a few building blocks can be arranged to provide stability against fluctuations. Our findings suggest that the transcriptional oscillator in yeast belongs to a new class of biological oscillators.

## Author Summary

Technologies such as gene arrays enable acquisition of large amounts of data on gene expression variations, which reveal the structures of gene regulatory networks that govern the metabolic and developmental machinery in the cell. We study a model of an oscillatory gene regulatory network that has been recently suggested to play an integral role in maintaining the cell cycle in yeast. The oscillator differs from other known biological and synthetic oscillatory networks in that it seems to rely on a long positive feedback loop. We show that the presence of certain stabilizing sub-networks can account for the robustness and the unusual architecture of this oscillator. Our modeling approach elucidates both the logical structure of the system and the importance of the timing of update events.

## Introduction

Cells have to operate reliably under internal and external noise in order to survive. Their robustness is partially a result of various signal-processing sub-networks called “motifs,” embedded in the transcriptional network of the cell that controls gene expression [1]–[5]. Such motifs are employed by the cell to produce reliable responses to internal and external signals: a negative auto-regulation motif decreases response time and increases robustness to noise [3], [6], [7]; a positive feedback generates bistability and thus can act as a switch [8]–[10]; a coherent feed-forward loop with OR logic acts like a capacitor, sustaining a high output when the input signal is transiently lost [11]; and an incoherent feed-forward loop allows adaptation to a sustained input signal [12].

It is known that combinations of some motifs such as positive and negative feedback loops, can generate stable cyclic behavior [1], [10], [13]–[19]. The exact mechanism underlying the oscillations may vary [20]–[22]. Two examples have been particularly well studied. In a negative feedback oscillator (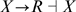), a sufficiently long time delay in the negative feedback loop makes the system repeatedly overshoot an unstable steady state [10], [13]. In an activator-inhibitor oscillator (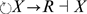), a positive feedback loop creates bistability and a negative feedback loop causes oscillations due to hysteresis [10], [13], [15], [16]. An important feature in these examples is the spontaneous activation of 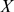, which is required to avoid collapse to a quiescent state. In a transcriptional oscillator, this corresponds to a constant input signal (due, for example, to a constitutive promoter) or positive auto-regulation sufficiently strong to cause levels of 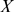 to rise to an active state as long as the inhibitor 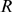 is not present.

To our knowledge, all models of biological oscillatory networks described in the literature, such as cyclin-cdc2 oscillations [23], [24], or circadian oscillations in *Drosophila* [25], require spontaneous activation to sustain the oscillations [1], [13], [20], [21]. This is also true for synthetic examples such as the repressilator [26], (in which all three genes have constitutive but repressible promoters), the *E. coli* predator-prey system [27], and the synthetic gene-metabolic oscillator [28]. The recently published transcriptional yeast (*Saccharomyces cerevisiae*) cell-cycle oscillator [29], however, does not seem to share this feature. The gene expression data suggest that this oscillator relies mainly on a sequence of activations on a long, slow positive feedback loop [29]–[31]. There does not appear to be an element in this transcriptional network that is activated spontaneously. Expression profiles also indicate that the period of the oscillator is very close, if not identical, to the time it takes for the wave of activations to cycle around the long positive feedback loop. Here, we show how it is possible to maintain stable oscillations within this architecture. We demonstrate that a slow positive feedback loop coupled to certain stabilizing motifs can sustain oscillations, and that a model of the transcriptional oscillator associated with the yeast cell-cycle works in this fashion.

Oscillator stability is conventionally studied in the context of a differential equation model [20], [21]. On the other hand, the essential organizing logic of regulatory networks can be studied much more easily using Boolean models [29], [32]–[40]. A drawback of the standard synchronous Boolean approach is that it does not permit the implementation of small perturbations, *i.e.*, noise, of the type that would result from stochastic fluctuations of the number of molecules of a given species or the rates of production of the various species involved. Indeed, synchronous Boolean models are known to produce many cyclic attractors that represent only marginally stable behavior, which disappear in the presence of noise [41], [42]. Here we take an intermediate approach that emphasizes the essential Boolean logic of the system within a continuous-time updating scheme that allows the modeling of small perturbations [41], [43]–[46]. We associate a time delay with each link in the network of regulatory interactions that determines the timing of activation and deactivation events. The stochastic fluctuations thus appear in our model as deviations of the delay times from their nominal values. Such models have been termed *autonomous* Boolean networks [47], [48] to distinguish them both from models based on synchronous or random asynchronous timing of updates and from Boolean Delay Equations [49], [50] that do not account for finite response times. The results presented here apply as well to appropriately constructed ordinary differential equation (ODE) models [46].

Regulatory networks based on the cyclin/CDK-centered view of the cell cycle [51] in *S. cerevisiae* [38] and *Schizosaccharomyces pombe* [52] have been studied previously using a synchronous Boolean framework. In those models, the intrinsic dynamics is not cyclic and the transition sequence corresponding to the cell cycle must be triggered by an external signal. We emphasize that the network we study is based on the recent experiments [29], [53] suggesting the existence of a self-sustaining transcriptional oscillator in yeast.

The rest of the paper is organized as follows. We first define the autonomous Boolean formalism and discuss the necessity for it. We then demonstrate that it is possible to construct a stable autonomous Boolean oscillator consisting of a long positive feedback loop with two stabilizing motifs added. This toy oscillator has topological features resembling the yeast cell-cycle oscillator. We then describe numerical experiments demonstrating that these features are the source of stability in the autonomous Boolean version of the network of Orlando *et al.* [29]. We close with a discussion of the implications of these findings. The details of the computer simulations are provided in the Methods section.

## Results

### Model

In an autonomous Boolean network (ABN), each node takes one of only two values at any given time: 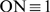 or 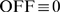. Updates are executed in continuous time as follows. When a node, 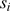, changes its state, it signals all the downstream nodes directly connected to its outputs. Each downstream node, 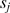, receives the signal after a time delay, 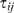, which is a real (not necessarily integer) value. When the signal is received, 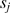 reevaluates its state according to its assigned Boolean function and adopts the resulting value, 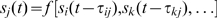. If the new value is different from its present value, a new signal is sent to its own downstream targets. Nodes do not update at externally dictated times, as in the synchronous model or various asynchronous versions. The update dynamics is determined by the timing of events, delays, and the topology of the network.

**Figure 1:**
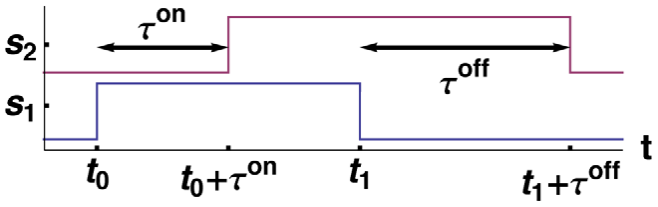
A pulse traveling on a cascade of two nodes, in which 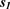 feeds 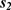. Both nodes are in OFF state at the beginning. 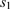 is turned on at 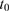 and turned off at 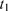. The pulse on 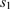 propagates to 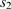 after a time delay. Asymmetric delays for the speeds of activation and deactivation events, 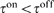, cause the pulse to grow as it propagates.

In principle, delays associated with activation (switch-on events), 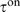, can be different from the ones associated with deactivation (switch-off events), 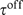, because of the different physical processes involved. The former characterizes multiple processes, including transcription, and translation, folding, post-translational modification, and spatial transport, while the latter can be attributed to degradation of mRNAs and transcription factors. The difference between 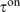 and 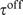 can cause a change in the duration of a pulse of transcriptional activity as it propagates down a chain of nodes [46]. Consider, for example, a simple cascade with two nodes, where output of 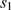 regulates 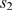. Suppose we turn 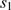 on manually at 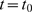 and turn it off at 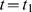, forming a pulse of width 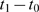, as shown in Figure 1. The rising edge of this pulse arrives at 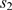 at 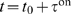 and the falling edge arrives at 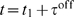. When 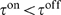, the initial pulse grows as it propagates (Figure 1) and if 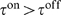, it shrinks.

Small perturbations due to stochastic fluctuations, or noise, can significantly alter the dynamics of a network and can be used as a mathematical tool for analyzing the stability of cycles. Noise is incorporated by taking the time delay associated with a switching event to be 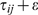, where the noise term, 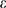 for each propagating signal is drawn at random from a uniform distribution on 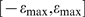 with 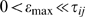.

For present purposes, we take the intrinsic delays 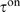 and 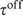 to be equal, allowing the noise to play a dominant role in determining which cycles are stable. The choice of 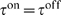 corresponds to the regime in which the asymmetry in propagation times is small compared to 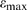, so that pulses grow or shrink according to the relative values of 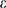 chosen for the leading and trailing edges.

In certain cases, the noisy dynamics can generate a pulse of negligibly small width, which we call a *spike* [41], [47], [48]. In the present context, a spike would correspond to arbitrarily fast build-up and degradation of transcripts and therefore is not realistic. We employ a short-pulse rejection mechanism in the simulations, discarding both pulses and dips with widths less than 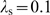 time unit. The Methods section below provides details of our computer simulation of ABNs.

### Oscillations on a Long Positive Feedback Loop

As mentioned above, the backbone of the oscillator in the network of interest is a positive feedback loop, also known as a *loop of copiers* or a *simple loop* because each node simply assumes the value of its input after some specified time delay. To demonstrate the need for a stabilization mechanism, we consider first the simple case of a loop of two copiers. We can assume without loss of generality that the two links have identical delays, 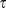. The network cycles between the 01 and 10 states when one node is initialized with a pulse of sufficiently large width. Setting that width equal to 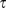 and setting the noise level to zero reproduces the dynamics of the synchronous Boolean case.

To test the stability of the 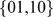 cycle, we apply arbitrarily small random perturbations: each time a signal propagates across a link, the delay is taken to be 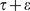, where 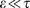 is a random number drawn from a distribution that is symmetric around zero. Each perturbation causes the pulse width to grow or shrink as explained above, so that the oscillation eventually collapses to either the 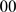 or 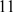 fixed point (stationary state). Thus the 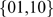 cycle is only marginally stable in the autonomous model and its apparent stability under synchronous updating is an artifact of that scheme.

### Stabilizing Motifs

**Figure 2:**
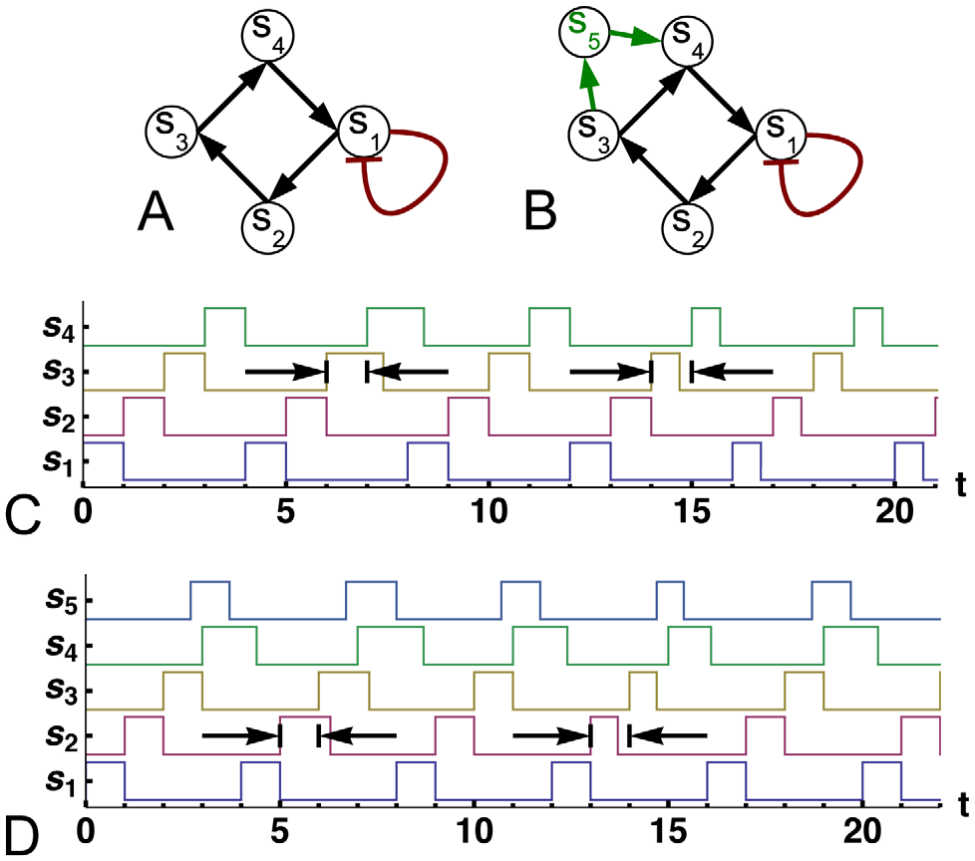
Dynamics on simple loops with stabilizing motifs. (A) A loop of copiers (a positive feedback loop) with an auto-repressive rectifier. (B) The same circuit as in (A) with an addition of a coherent feed-forward loop that can act as a grower with OR logic. (C,D) Dynamics on the circuits (A) and (B), respectively. Both circuits were initialized by a pulse of unit width on 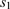 at 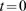, and all nodes were assumed to be OFF for 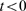. First cycles in both graphs show the time series for the single-pulse attractor, to which this initial condition leads. Circuits were perturbed on the second and the fourth cycles to demonstrate how stabilizing motifs operate. Arrows indicate unperturbed pulse widths. The pulse-growing perturbation in (C) is filtered by the rectifier and the dynamics recovers as it returns to the original attractor in the third cycle. However, the pulse-shrinking perturbation in this circuit pushes the dynamics to a marginally-stable cycle as it cannot be corrected only by a rectifier. A grower-rectifier combination in (B) can filter both pulse-shrinking and -growing perturbations: dynamics recover in the third and fifth cycles in (D). Note that the pulse on the output of the grower, 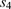, is wider than that on the input, 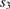. All 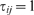 except 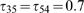 in (B).

We identify two classes of motifs, [1], [2], [4], which we call *rectifiers* and *growers*, that can correct small perturbations to the timing of the updates and stabilize cycles on an autonomous loop of copiers. A rectifier imposes an upper limit on the width of the pulse traveling on the positive feedback loop. The simplest example of a rectifier is auto-repression (Figure 2A), which cuts long pulses down to a width equal to the delay on the auto-repressive link, 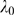, and lets short pulses pass through unaffected [46]. Small perturbations that cause the pulse width to exceed 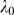 will be filtered by this motif as seen in Figure 2C. An incoherent feed-forward loop of type 1 (I1-FFL in the notation of [1]), and a negative feedback containing more than one node can also function as rectifiers.

Grower motifs increase the duration of a pulse by a constant amount, but do not adjust them to a particular value. One example is the coherent feed-forward loop with OR logic (C1-FFL-OR [1], [2]) shown in Figure 2B. This motif grows pulses by transmitting the input pulse of width 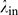 from 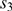 to 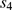 through two paths with time delays that differ by 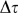. The slower path sustains the output, producing a pulse of width 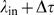, assuming 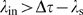. (If the condition is not met, two pulses will be generated.) A diamond motif [1] with OR logic, in which both paths connecting the input to the output contain an intermediate node, functions in the same manner. We also note that both C1-FFL and the diamond motifs function as *shrinkers* when their output is an AND gate, shrinking the input pulse by 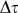 or destroying it completely.

### Combining a Grower and a Rectifier to Sustain Stable Oscillations

A rectifier cannot prevent the collapse to the all-OFF state and a grower alone inserted in a loop will keep growing the pulse until the all-ON attractor is reached. The two motifs working in tandem (Figure 2B), however, can act as a stabilizing module for cyclic attractors, as seen in Figure 2D: both pulse-growing and pulse-shrinking perturbations are filtered because the grower-rectifier combination resets the pulse width to 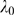 after each cycle. Such a network can sustain stable oscillations that have been started with an external signal. The two motifs will be incompatible if 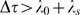 because the grower will generate two pulses from each rectified pulse.

We note that there is no simple motif that acts as a low-pass rectifier, allowing long pulses to pass unaffected while boosting short pulse widths up to a specified value. Thus the shrinker motif is of limited use for stabilizing oscillations. Furthermore, a grower-shrinker combination cannot be a stabilizer as it simply acts either as an overall grower or an overall shrinker.

If one allows 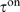 and 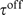 to be different, a pulse may grow or shrink as it travels around a simple loop. When 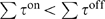 for the links in the loop, we have a source of “intrinsic growth” that may render a grower motif unnecessary, or just assist the grower in restoring pulse widths more rapidly. In fact, it has been shown using an ODE model with time delays that when switch-on events propagate faster than switch-off events, an auto-repressive link can by itself create a stable cycle on a loop of copiers [46]. Similarly, when 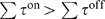 along the loop, a pulse will shrink as it propagates. Stabilization in the presence of intrinsic shrinkage requires a grower regardless of the noise level. We do not consider intrinsic growth or shrinkage here, focusing instead on cases where stochastic effects (noise) dominate over the intrinsic effects. Also, we consider only the stabilization of *single-pulse cycles*, in which each node along the loop (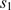 through 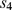) turns on and off exactly once per cycle time, which we define as the time required for a single signal to propagate around the loop once. For a simple loop, the cycle time is equal to the sum of the delays, but for more complex circuits, it can depend on the pulse width.

### An Oscillator without Frustration

A crucial feature of the oscillator architecture under consideration here (Figure 2B) is that it does *not* rely on any constitutive input or positive auto-regulation. Consider, for example, the model of circadian oscillations in *Drosophila* [13], [21], [25], which contains one protein, PER, whose biphosphorylated form represses its own transcription. It is assumed that *Per* mRNA is transcribed at the maximum rate in the absence of biphosphorylated nuclear PER, thereby building up spontaneously. Such an oscillator can be represented as a simple negative feedback loop, *Per* 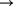
PER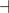 *Per* with a long time delay on the repressive link. A Boolean model of the oscillator can be constructed by assigning a NOT function to *Per* indicating that it builds up spontaneously, but only in the absence of PER; and a COPY function to PER as it is produced only in the presence of *Per*. This model has a cycle containing all four states of the circuit, 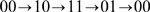.

From the Boolean perspective, the underlying principle for these oscillations is the impossibility of satisfying all the Boolean functions simultaneously, as the combination of an inverter and a copier creates *frustration* [42]. For this reason, we refer to the Boolean versions of such oscillators, which have no fixed points, as *frustration oscillators*. The oscillator we propose in Figure 2B, however, has the all-OFF fixed point attractor; there is no frustration in its logic. It therefore belongs to a different class that involves a stable transmission of a pulse on a loop of copiers, *i.e.*, a positive feedback loop. We refer to these as *transmission oscillators*.

### The Yeast Cell-Cycle Oscillator

**Figure 3:**
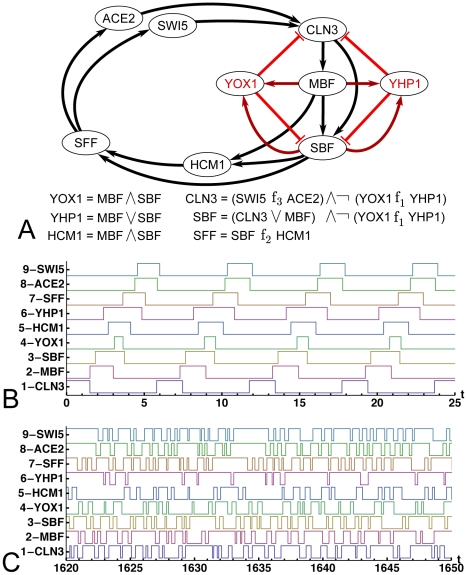
Yeast cell-cycle network and the autonomous Boolean dynamics on it. (A) The network diagram and the regulatory logic template for the yeast cell-cycle oscillator. Single-input nodes are copiers; logic functions for multi-input nodes are indicated. 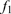, 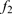, and 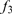, may each be either AND or OR as listed in Table 1 (B) A typical realization of the autonomous Boolean model with random delays for the network that sustains a stable single-pulse oscillation. (C) A realization in which the network generates complex oscillations.

The recently published cell-cycle oscillator network in yeast consists of nineteen interactions between eight transcription factors and one cyclin, CLN3, which was used as a proxy for currently unidentified transcription factors that complete the circuit (Figure 3A) [29], [30], [53]. The regulatory logic functions of the multi-input nodes are not known. This oscillator was studied using a synchronous Boolean model with eight different “biologically interpretable” logic configurations for the network given in Figure 3A and Table 1 [29]. Each logic configuration was found to support at most two out of the three possible cycles in addition to the all-OFF fixed point. All three cycles match the sequential order of the expression of the transcription factors. We emphasize here, however, that these features may only be artifacts of the synchronous update scheme and their stability requires further investigation.

**Table 1:** Regulatory logic selections and the simulation results for the yeast cell-cycle oscillator.

Cfg.	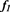	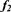	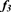	Complex	PSP
1	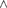	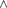	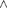	0	0
2	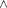	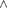	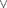	0.05	0.13
3	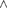	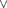	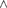	0.22	0.3
4	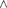	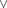	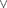	0.65	0.35
5	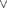	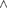	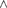	0	0
6	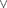	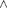	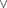	0.01	0.04
7	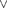	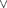	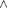	0.05	0.14
8	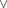	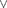	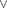	0.58	0.3

All logic configurations tested, which employ different Boolean function combinations for repression, SFF and CLN3. Last two columns show the fraction of realizations that exhibit complex (includes multi-pulse) and periodic single-pulse oscillations in an ensemble of 10000 realizations of the network with quenched random delays. The simulations employ noise and short-pulse rejection. Standard errors in the fractions quoted are of 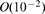, which were estimated by pooling the results into bins of 500 realizations each.

This version of the yeast cell-cycle oscillator is a complex network that does not seem to be a frustration oscillator. Expression profiles of transcription factors suggest that sequential activations are triggered by immediate upstream regulators in the network [29]. Therefore, the oscillations are unlikely to be driven by a frustration oscillator that is either a part of or coupled to the circuit. Several intertwined feed-forward and negative feedback motifs in the network suggest that a grower-rectifier combination may be at play in stabilizing the oscillations. Specifically, we hypothesize that this network is a simple loop consisting of CLN3, SBF, SFF, and ACE2 or SWI5 (since this is the loop of copiers with the least number of links), and all other nodes conspire to provide stabilizing motifs. We use computer simulations to test this hypothesis. Briefly, we assign random delays to each link and start the network by manually turning CLN3 on then off. The distribution we choose for the delays roughly captures the variation in delays seen in the experiments [29]. A broader distribution would not qualitatively change the results. The details of the simulations are described in Methods.

#### Dynamics

For all logic configurations tested, the long-term behavior we observe in the simulations can be classified in three categories: a) *Collapse*: the network settles onto all-OFF fixed point. There is no all-ON fixed point because all of the logic possibilities for SBF in Table 1 require that SBF turn off if YOX1 and YHP1 are both on. b) *Periodic single-pulse (PSP) oscillations*: the network maintains a stable single-pulse oscillation on all nodes (Figure 3B). c) *Multi-Pulse or Complex oscillations*: oscillations are either periodic multi-pulse, with some nodes pulsating more than once per cycle time, or complex, with strong variations in pulse widths and no identifiable underlying period (Figure 3C) [49], [50]. Determining the character of complex oscillations is outside of the scope of this paper. We consider the regime of complex oscillations to be biologically unrealistic and limit our attention from here on to the periodic single-pulse oscillations. The fraction of realizations that yield PSP and complex oscillations is given for each logic configuration in Table 1.

#### Testing for presence of stabilizing motifs

We hypothesize that the stability of the yeast cell-cycle must be due to a grower-rectifier combination. The rectifier candidates in the network are the motifs that contain repressors YOX1 and YHP1. For the given logic configurations, YHP1 is the repressor in three intertwined rectifiers. YOX1 may also contribute to rectification of the pulse width, but the rectifying motif is complicated by the fact that YOX1 is activated only when both MBF and SBF are ON. Both YHP1 and YOX1 are required for repression for logic configurations 1–4. The particular motif or the motif combination that limits the pulse width depends on the response delays. Consequently, some rectifiers will be redundant in some realizations of the model.

Identifying the specific locus of growth and rectification in a typical network realization is very difficult. The grower and rectifier motifs in the network can be intertwined, and any specific motif can be functional or redundant in a given realization. We test for the presence of growth and rectification functions without identifying the actual grower and rectifier motifs, using the following reasoning and numerical procedure.

Any functional rectifier in the yeast cell-cycle oscillator model has to contain YOX1 and/or YHP1, as they are the only repressors. Removal of YOX1 and YHP1 will disable all possible rectifiers in the network. As mentioned above, a loop with a grower but no rectifiers keeps growing pulses and eventually reaches the all-ON attractor. If it is true that the stabilization of oscillations is due to rectification and growth, then all stably oscillating realizations of the model should collapse onto the all-ON attractor when YOX1 and YHP1 are removed. In other words, the net effect of all motifs decorating the long positive feedback loop that do not contain repressors must be growth.

In order to see whether there are functional grower-rectifier combinations in the yeast cell-cycle oscillator, we selected from an ensemble of 10000 network realizations only those for which a single-pulse oscillation endures indefinitely, and removed the repressors after the network settled onto the attractor. Specifically, we turned the noise off after 90000 updates, ran the network for another 35000 updates without noise, and checked for periodicity. If the oscillations showed a PSP character, we “knocked out” the repressors while the oscillator was running and observed the dynamics to see whether the pulse grows from one cycle to the next.

The simulations show that for all eight logic configurations, every realization that produces stable periodic single-pulse oscillations does indeed go to the all-ON attractor when YOX1 and YHP1 are knocked out. The pulse width increases on each cycle after repressors are disabled.

The pulse growth observed in the runs with disabled repressors is not due to noise. First, convergence onto the all-ON attractor is more rapid than the amplitude of the noise would permit. Second, the noise can both grow and shrink the pulse, leading to a one-dimensional random walk of the pulse width. For the considered initial pulse width, the network would be much more likely to reach the all-OFF attractor than the all-ON state if the dynamics did not provide for pulse growth. We find that none of the selected realizations of the yeast cell-cycle model collapses to the all-OFF attractor when YOX1 and YHP1 are removed, showing that noise is not the source of pulse growth in these models.

The results indicate that all realizations of the autonomous model of the yeast cell-cycle oscillator that generate stable periodic single-pulse oscillations contain functional growers and rectifiers.

## Discussion

We have shown using an autonomous Boolean model, that a long positive feedback loop can be turned into a stable oscillator with the addition of two stabilizing motifs that can correct fluctuations in the pulse width (the duration of activity of each node in the network): a rectifier involving a repressor that limits the width of the traveling pulse, and a grower that lengthens the duration of a pulse so that it cannot shrink and disappear. In combination, a grower and a rectifier ensure that the pulse width returns to the same value after each cycle. The recently published yeast cell-cycle oscillator [29] has a structure built around a long positive feedback loop, on which waves of activation events propagate. Numerical simulations of eight different logic configurations and multiple realizations of randomly assigned time delays revealed the presence of grower and rectifier functions in this network. To our knowledge, there is no other biological oscillator model described in the literature that relies on a long, slow positive feedback loop. We note that a proposed cell cycle network for *Caulobacter crescentus* [54] has a structure reminiscent of that of yeast, but no dynamical model of it has yet been reported.

Previous synchronous Boolean models of *Drosophila* segmentation network [39], or cyclin/CDK-based cell-cycle networks of *S. pombe* [52] and *S. cerevisiae* [38] predicted essential features of the robust dynamics of these networks [55]. We have demonstrated that the autonomous Boolean framework can be used to further study such problems, since it addresses important elements of the regulatory dynamics associated with the timing of updates and the effects of stochastic fluctuations. We note that ABNs have also been used recently for analyzing chaos and the stability of periodic orbits in digital electronic oscillators [47], [48].

Our results also point to a drawback of fully asynchronous Boolean models: a stable cycle in a continuous-time system such as that of Figure 2D would not be observed in an asynchronous model. In asynchronous models, cycles generated by loops containing an even number of inverters cannot be sustained [42] because there always exists a sequence of updates that leads to the fixed point state. We have shown, however, that when appropriate motifs are present, the autonomous rules for determining the order in which nodes are updated never permit evolution to the fixed point even in the presence of a substantial level of noise. In analyzing the dynamics of gene networks containing feedback loops, it is therefore important to take into account timing information associated with signal propagation. For gene networks containing feedback loops, results from discrete-time Boolean models (both synchronous and asynchronous) should be interpreted with care.

The stability of the oscillations we have observed is not an artifact of the autonomous Boolean model. The presented results are qualitatively compatible with ODE analogs involving explicit time delays [56]. An ODE model of a similar system with explicit time delays has already been shown to exhibit stable oscillations very similar to our Boolean idealization when synthesis rates, Hill coefficients, and time delays are large enough [46]. Our own preliminary studies indicate that it is also possible to construct an ODE model of a transmission oscillator without explicit time delays by selecting appropriate parameters for the stabilizing motifs.

## Methods

To simulate the dynamics of an autonomous Boolean network, we use an event-driven code. A time-ordered event queue is established, in which each event represents the switching of an input at a specified node. Each time an event is processed that results in the switching of a node, events are added to the queue according to the time delay associated with each output link from that node. After each update of a node, we check to see whether it creates a short pulse that should be rejected. If so, the queue is purged of all events derived from the leading and trailing edges of the pulse. To avoid causality problems coming from propagation of a switching event that is later rejected, we choose the maximum noise amplitude, 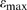, to be less than half of the short-pulse rejection time (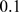 time unit).

To reveal the structure of the yeast cell-cycle oscillator, we study numerical simulations of autonomous Boolean versions of the network with the logic choices in Reference [29] and different randomly selected sets of time delays. For each logic configuration, we generate an ensemble of 10000 networks with quenched random delays on each link. Delays were chosen from a uniform distribution between 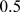 and 2 time units. The system was initialized by turning CLN3 on at 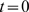 and turning it off at 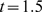, while other nodes were OFF. All nodes were assumed to be OFF for 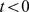. To simulate noise, a random value selected from a uniform distribution on the interval 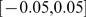, was added to the delay associated with each update. We are interested in the stability of a particular cycle, so we have chosen an initial condition that is very likely to lie in the basin of attraction of that cycle (if the cycle exists). A different initial condition, turning CLN3 on at 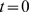 and letting a repressor turn it off, yields roughly the same statistics reported in Table 1. We do not test the network's robustness to general changes in the initial condition [38], [39], [57], [58].

We run simulations up to 125000 updates with noise turned on between the 800th and 90000th updates in order to eliminate marginally stable oscillations. For single-pulse oscillations, this typically translates into a runtime of 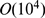 time units under noise. Periodic single-pulse oscillations that survive this long with noise present are highly likely to be stable attractors. An oscillation is considered to be PSP if pulse widths on two consecutive cycles differ by less than 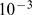 time unit on each node. We do not check whether all nodes turn on and off once per cycle time, *i.e.*, whether the cycle is a single-pulse or a dual-pulse with identical pulse widths. However, we never observed the latter in the inspected realizations and believe that it is very unlikely to occur in this circuit.

The numbers of oscillating realizations differ in the different logic configurations for two reasons. First, an FFL or diamond motif operates as a grower with OR logic and as a shrinker with AND logic. When two logic configurations differ only by the selection of 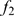 or 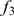, the one with OR logic always has a larger number of oscillating networks. In configurations 1 and 5, both SFF and CLN3 are AND gates, so the motifs they belong to will act as shrinkers. The existence of two shrinkers in the network should make oscillations very unlikely, and indeed we find that all realizations in both configurations collapse on the all-OFF attractor. On the other hand, configurations that contain a larger number of AND logic for 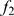 and 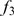 generate mostly periodic single-pulse oscillations and fewer complex ones. The second reason for the difference in the number of oscillating networks is the logic, 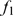, of the repressors. The 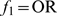 case gives a smaller total number of oscillating realizations than 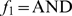.
